# Racial-ethnic disparities in potentially preventable complications after cesarean delivery in Maryland: an observational cohort study

**DOI:** 10.1186/s12884-022-04818-5

**Published:** 2022-06-16

**Authors:** Allison Lankford, Laura Roland, Christopher Jackson, Jonathan Chow, Ryan Keneally, Amanda Jackson, Rundell Douglas, Jeffrey Berger, Michael Mazzeffi

**Affiliations:** 1 Department of Obstetrics and Gynecology, University of Maryland School of Medicine, Baltimore, MD USA; 2 Department of Anesthesiology and Critical Care Medicine, George Washington University School of Medicine and Health Sciences, Washington DC, 20037 USA; 3 Department of Obstetrics and Gynecology, Walter Reed National Medical Center, Bethesda, MD USA; 4 George Washington University Milken Institute School of Public Health, Washington DC, USA

**Keywords:** Cesarean delivery, Obstetrics, Healthcare quality, Disparities

## Abstract

**Background:**

Potentially preventable complications are monitored as part of the Maryland Hospital Acquired Conditions Program and are used to adjust hospital reimbursement. Few studies have evaluated racial-ethnic disparities in potentially preventable complications.

Our study objective was to explore whether racial-ethnic disparities in potentially preventable complications after Cesarean delivery exist in Maryland.

**Methods:**

We performed a retrospective observational cohort study using data from the Maryland Health Services Cost Review Commission database. All patients having Cesarean delivery, who had race-ethnicity data between fiscal years 2016 and 2020 were included. Multivariable logistic regression modeling was performed to estimate risk-adjusted odds of having a potentially preventable complication in patients of different race-ethnicity.

**Results:**

There were 101,608 patients who had Cesarean delivery in 33 hospitals during the study period and met study inclusion criteria. Among them, 1,772 patients (1.7%), experienced at least one potentially preventable complication. Patients who had a potentially preventable complication were older, had higher admission severity of illness, and had more government insurance. They also had more chronic hypertension and pre-eclampsia (both *P*<0.001). Median length of hospital stay was longer in patients who had a potentially preventable complications (4 days vs. 3 days, *P*<0.001) and median hospital charges were approximately $4,600 dollars higher, (*P*<0.001). The odds of having a potential preventable complication differed significantly by race-ethnicity group (*P*=0.05). Hispanic patients and Non-Hispanic Black patients had higher risk-adjusted odds of having a potentially preventable complication compared to Non-Hispanic White patients, OR=1.26 (95% CI=1.05 to 1.52) and OR=1.17 (95% CI=1.03 to 1.33) respectively.

**Conclusions:**

In Maryland a small percentage of patients undergoing Cesarean delivery experienced a potentially preventable complication with Hispanic and Non-Hispanic Black patients disproportionately impacted. Continued efforts are needed to reduce potentially preventable complications and obstetric disparities in Maryland.

**Supplementary Information:**

The online version contains supplementary material available at 10.1186/s12884-022-04818-5.

## Background

Caesarean delivery (CD) is the most common surgical procedure performed in the United States with approximately 30% of all pregnant patients and over one million patients per year having CD [1]. Up to 20% of patients who have CD experience postoperative complications with the most common complications being postpartum hemorrhage, wound infection, urinary tract infection, endometritis, surgical site infection, and reoperation for bleeding or infection [2]. Prior studies suggest that between 2% and 15% of women have surgical site infection after CD, which increases hospital length of stay and morbidity [3, 4].

Postoperative complications occur more frequently in Non-Hispanic Black and Hispanic patients in the United States. In a prior study that used administrative data from New York state, and included over one million surgical patients, Non-Hispanic Black patients had 18% increased odds of postoperative complications after controlling for preoperative risk [5]. In an observational study that included over 40,000 bariatric surgery patients, Non-Hispanic Black patients were 72% more likely to experience postoperative complications, including hospital readmission, when compared to Non-Hispanic White patients [6].

Potentially preventable complication (PPCs) are tracked by the Maryland Health Services Cost Review Commission (HSCRC) as part of the Maryland Hospital Acquired Conditions Program, which began in 2011. PPCs are identified using statewide administrative data and an algorithm (PPC grouper) developed by 3M Health Information Systems (Salt Lake City, UT USA). PPC rates for individual hospitals are calculated and used to adjust hospital reimbursement when a hospital’s PPC rate is above the statewide mean. There are currently fifty-seven diagnoses, which are considered to be potentially preventable, and are associated with patient harm. Examples of PPCs include deep venous thrombosis, pulmonary embolism, and surgical site infection.

To our knowledge and based upon our literature review, there are no studies exploring racial-ethnic disparities in PPCs after CD. The aim of our study was to explore whether there were racial-ethnic disparities in PPCs after CD in Maryland. We hypothesized that Non-Hispanic Black patients, Non-Hispanic Asian patients, Non-Hispanic patients of other races, and Hispanic patients, would have higher rates of PPCs compared to Non-Hispanic White patients.

## Methods

### Patients

Patients who underwent elective, urgent, or emergent CD for any indication in Maryland between fiscal years 2016 and 2020 (July 1^st^ 2015 and June 30^th^ 2020) were identified for inclusion using the HSCRC database, which contains data for all hospitalized patients in Maryland. The study period was selected because it represented a period of consistent healthcare policy within the state, where the global budget revenue program was in place and PPCs were recorded as a quality metric. Medicare severity diagnosis related group (DRG) codes were used to identify patients who underwent CD. The following Medicare DRGs were used: 783, 784, 785, 786, 787, and 788. Patients were excluded from the analysis if they were missing race-ethnicity data.

### Patient Variables

Demographics including age group, race, and ethnicity were collected for all patients. Race-ethnicity data were based on administrative data officially submitted to the HSCRC by Maryland hospitals. Race was categorized as Non-Hispanic White, Non-Hispanic Black, Non-Hispanic Asian, Non-Hispanic patients of other races, and Hispanic. Non-Hispanic White patients were considered as the reference group because they were hypothesized to have the lowest PPC incidence. Further, we collected marital status and primary insurer (government, commercial, or other). Medical data included all patient refined (APR) severity of illness at hospital admission, chronic hypertension, pre-eclampsia, diabetes mellitus, gestational diabetes, prior CD, and preterm delivery. Medical diagnoses were based on international classification of disease (ICD) 9 and 10 codes. Finally, we collected data on hospital length of stay, total hospital charges, and unplanned hospital readmission within thirty days.

### PPCs

The HSCRC identifies PPCs using proprietary software developed by 3M Health Information Systems (Salt Lake City, UT USA). Secondary diagnoses that are not present at hospital admission are used to identify specific complications using ICD-9 or 10 codes. PPC methodology was originally developed using administrative data from California in the late 1990s and has been refined over time. Patients in our study had PPCs identified using PPC grouper versions 36.0 and 37.0. Supplemental Table 1 lists the specific PPCs that were recorded with PPC grouper 36.0 and 37.0. PPC categories included extreme complications (e.g. cardiac arrest, shock), infectious complications (e.g. surgical site infection, urinary tract infection), and cardiovascular and respiratory complications (e.g. congestive heart failure, deep venous thrombosis, acute pulmonary edema).

### Primary outcome

The study’s primary outcome was occurrence of any PPC during hospitalization. Secondary outcomes were total hospital charges, length of hospital stay, and unplanned hospital readmission.

### Statistical analysis

Statistical analysis was performed using SAS 9.4 (SAS Corporation, Cary NC, USA). Continuous patient variables were summarized as median and interquartile range (skewed variables) or mean ± standard deviation (normal variables). Categorical variables were summarized as the number and percentage of patients. Patient characteristics were compared between patients who did and did not have a PPC using the Wilcoxon Rank Sum test, Student’s T test, Pearson’s Chi-Squared test, or Fisher’s exact test as appropriate. PPC incidence was calculated for each hospital in the state and was plotted on a Figure along with the hospital’s total CD volume. The number of PPCs in each race-ethnicity group was compared using Fisher’s exact test. Additionally, we created scatterplots with fitted regression lines showing unadjusted relationships between PPC incidence and the percentage of patients from a race-ethnicity group in each hospital.

To explore whether race-ethnicity had an independent association with PPCs, we performed multivariable logistic regression, where occurrence of any PPC was modeled as the dependent variable. Independent variables included in the model were race-ethnicity group and variables that were thought to be a priori confounders including age group, primary payer, year, hospital, prior CD, chronic hypertension, diabetes mellitus, pre-eclampsia, admission APR severity of illness, and pre-term delivery. Odds ratios with 95% confidence intervals were calculated for all independent variables in the logistic regression model. Model diagnostics included goodness of fit testing and area under the receiver operating characteristic curve analysis. The Strengthening the reporting of observational studies in epidemiology checklist was referenced and completed in preparing the manuscript.

### Ethics approval

The George Washington University institutional review board approved the study, determined it to be exempt as non-human subjects research, and waived the requirement for written informed consent. All methods were carried out in accordance with relevant guidelines and regulations.

## Results

**Fig. 1:**
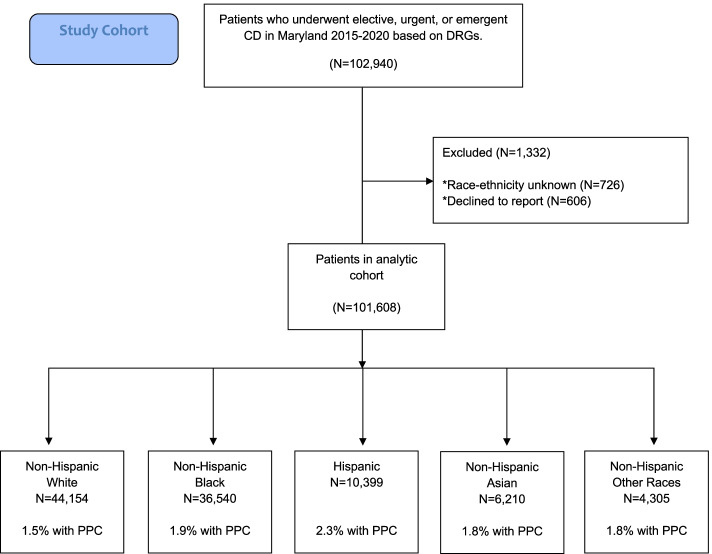
Figure shows study enrollment and exclusions

Figure 1 shows the study enrollment diagram. A total of 102,940 patients had CD in 33 hospitals during the five-year study period. One thousand three hundred thirty-two patients were excluded because of missing race-ethnicity data and 101,608 CD patients were analyzed. Among them, 1,772 patients (1.7%), experienced at least one PPC. Table 1 lists characteristics of patients who had a PPC and those who did not. Patients who had PPCs were older and were less likely to be married. Patients who had PPCs were also less likely to have commercial insurance and had higher admission severity of illness. Specifically, patients who had PPCs had a two-fold higher prevalence of chronic hypertension and pre-eclampsia (both *P*<0.001). Median length of hospital stay was longer in patients with PPCs (4 days vs. 3 days, *P*<0.001), median hospital charges were approximately $4,600 dollars higher (*P*<0.001), and unplanned hospital readmissions were more frequent (4.6% vs. 1.6%, *P*<0.001).

**Table 1:** Patient characteristics

Variable	No PPC ***N***=99836	PPC ***N***=1772	***P*** value
Age group			
≤ 19	2313 (2.3)	59 (3.3)	<0.001
20-24	12015 (12.0)	215 (12.1)	
25-29	24852 (24.9)	411 (23.2)	
30-34	33119 (33.2)	550 (31.0)	
35-39	21453 (21.5)	391 (22.1)	
40-44	5489 (5.5)	121 (6.8)	
≥ 45	595 (0.6)	25 (1.5)	
Race-ethnicity group			
Non-Hispanic White	43495 (43.6)	659 (37.2)	<0.001
Non-Hispanic Black	35856 (35.9)	684 (38.6)	
Non-Hispanic Asian	6099 (6.1)	111 (6.3)	
Non-Hispanic other	4226 (4.2)	79 (4.4)	
Hispanic	10160 (10.2)	239 (13.5)	
Marital status			
Single	39895 (40.0)	760 (42.9)	<0.001
Married	56567 (56.7)	933 (52.7)	
Separated or divorced	1808 (1.7)	39 (2.1)	
Widow	105 (0.1)	0 (0)	
Not reported	1459 (1.5)	40 (2.3)	
Primary payer			
Government	43855 (43.9)	876 (49.4)	<0.001
Commercial insurance	54688 (54.8)	876 (49.4)	
Other	1293 (1.3)	20 (1.2)	
Admission APR severity of illness			
Mild	55375 (55.5)	560 (31.6)	<0.001
Moderate	33072 (33.1)	583 (32.9)	
Severe	10891 (10.9)	517 (29.2)	
Extreme	498 (0.5)	112 (6.3)	
Chronic hypertension	1980 (2.0)	78 (4.4)	<0.001
Pre-eclampsia	8620 (8.6)	307 (17.3)	<0.001
Diabetes mellitus	2062 (2.1)	59 (3.3)	<0.001
Gestational diabetes	10593 (10.6)	176 (9.9)	0.36
Prior Cesarean delivery	16692 (16.7)	283 (16.0)	0.40
Preterm delivery	2936 (2.9)	101 (5.7)	<0.001
Total length of hospital stay	3 [3, 4]	4 [3, 5]	<0.001
Total hospital charges ($)	8469 [6440, 11642]	13111 [9152, 20423]	<0.001
Unplanned hospital readmission	1635 (1.6)	81 (4.6)	<0.001

*APR *all patient refined, *PPC *potentially preventable complication

**Fig. 2:**
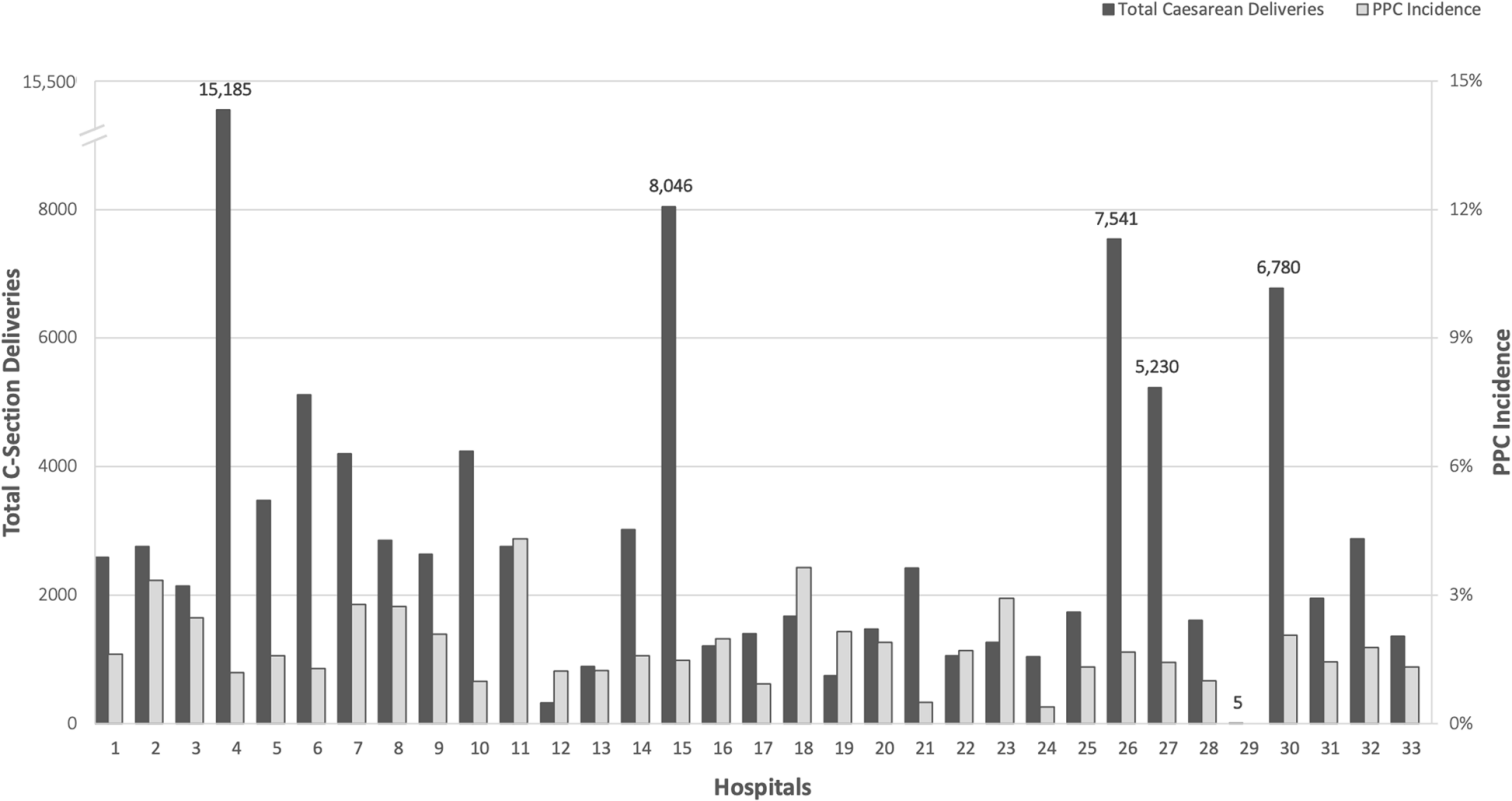
Figure shows five-year PPC incidence for individual hospitals that performed Caesarean delivery in Maryland from fiscal year 2016 to 2020

**Fig. 3:**
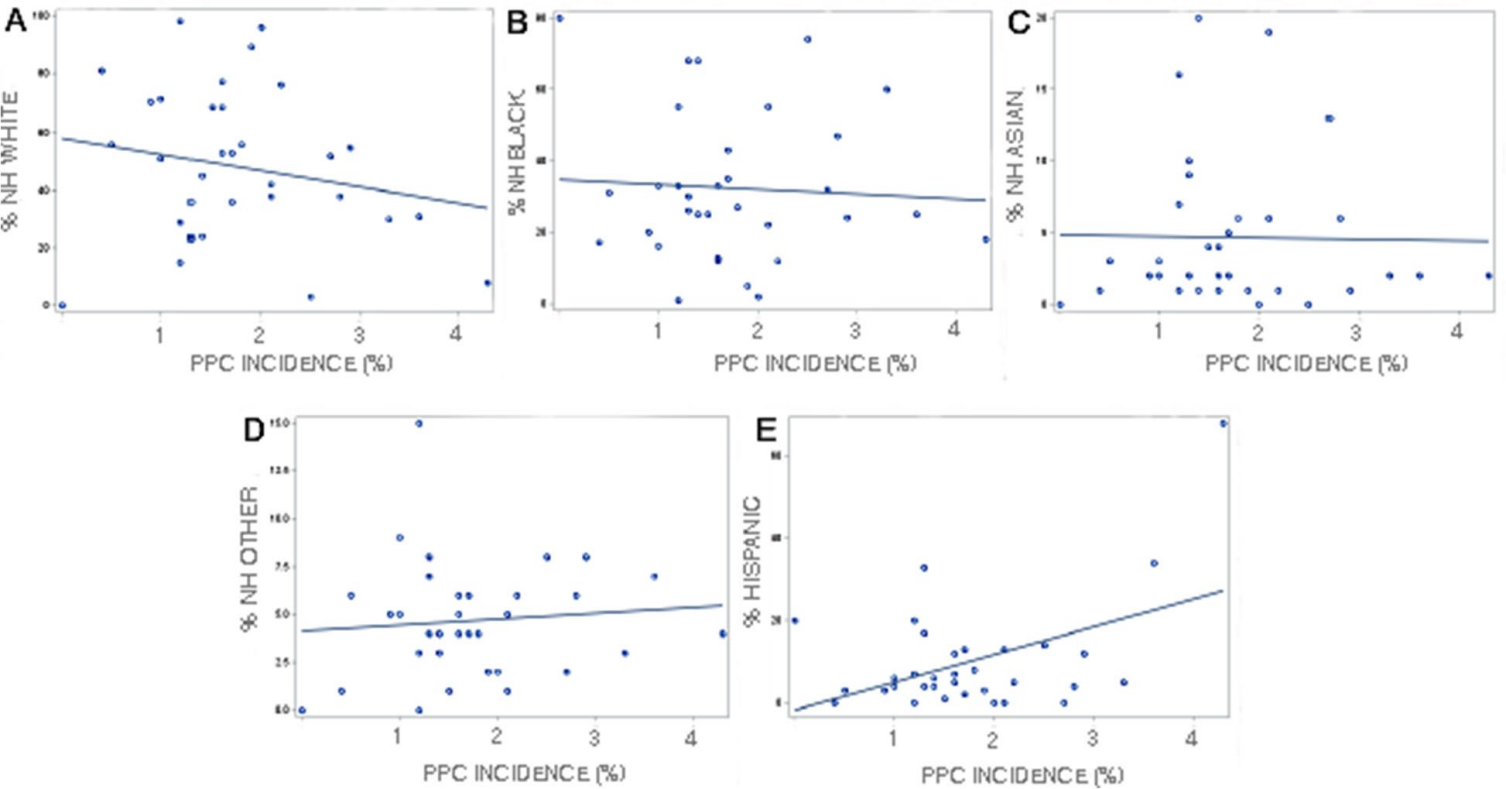
Figure shows unadjusted relationships between race-ethnicity group composition and PPC incidence in Maryland obstetric hospitals

Figure 2 shows the PPC incidence for individual hospitals in Maryland, which varied from 0% to 4.3%. Table 2 lists select PPCs of interest and their incidences. The majority of patients who had a PPC (84.1%), had a single event, while 5.5% of patients had 3 or more PPCs. Infectious complications including “major puerperal infection” and “reopening of surgical site for infection” were two of the most common PPCs. Table 3 shows the number of PPCs by race-ethnicity group. The number of PPCs differed significantly between groups (*P*<0.001) and the largest difference occurred in patients who had a single PPC. Figure 3 shows unadjusted relationships between race-ethnicity group and PPC incidence in Maryland hospitals.

**Table 2:** Select potentially preventable complications in cohort

Variable	N (%)
Number of PPCs per patient	
1	1490 (1.5)
2	185 (0.2)
3 or more	97 (0.09)
Select PPC incidences	
*Neurologic*	
Stroke or intracranial hemorrhage	5 (0.005)
*Respiratory*	
Acute pulmonary edema and respiratory failure without ventilation	86 (0.08)
Acute pulmonary edema and respiratory failure with ventilation	14 (0.01)
Aspiration pneumonia	7 (0.007)
Pulmonary embolism	6 (0.006)
*Cardiovascular*	
Cardiac arrest	7 (0.007)
Deep venous thrombosis	4 (0.004)
*Infectious*	
Clostridium difficile colitis	4 (0.004)
Sepsis	19 (0.02)
Major puerperal infection	102 (0.1)
Reopening of surgical site for infection	60 (0.06)
Urinary tract infection	8 (0.008)
Catheter associated-urinary tract infection	3 (0.003)
*Renal*	
Renal failure requiring dialysis	1 (0.001)
*Hematologic*	
Perioperative hemorrhage without hemorrhage control procedure	42 (0.04)
Perioperative hemorrhage with hemorrhage control procedure	16 (0.02)
*Obstetric*	
Medical and anesthesia obstetric complications	391 (0.4)

*2205 total PPCs in 1772 patients

*PPC *potentially preventable complication

**Table 3:** Number of potentially preventable complications by race-ethnicity group

Number of PPCs	Non-Hispanic White	Non-Hispanic Black	Non-Hispanic Asian	Non-Hispanic other races	Hispanic
0	43495 (98.5)	35856 (98.1)	6099 (98.2)	4226 (98.2)	10160 (97.7)
1	569 (1.3)	556 (1.5)	87 (1.4)	67 (1.6)	211 (2.0)
2	62 (0.1)	85 (0.2)	12 (0.2)	7 (0.1)	19 (0.2)
3 or more	28 (0.1)	43 (0.2)	12 (0.2)	5 (0.1)	9 (0.1)

*P<0.001 for the comparison between groups

*PPC* potentially preventable complication

Table 4 lists the results of the multivariable logistic regression model. The risk-adjusted odds of having a PPC were significantly different for patients from different race-ethnicity groups (*P*=0.05). The AUROC for the multivariable model was 0.72 suggesting good discrimination. Hispanic and Non-Hispanic Black patients had higher risk-adjusted odds of having a PPC compared to Non-Hispanic White patients, OR=1.26 (95% CI=1.05 to 1.52) and OR=1.17 (95% CI=1.03 to 1.33) respectively. Other variables that had a significant association with PPC occurrence included age, year, prior CD, pre-eclampsia, admission APR severity of illness, and hospital (all *P*<0.05).

**Table 4:** Multivariable regression analysis for occurrence of any potentially preventable complication

Variable	Odds ratio with 95% CI	***P*** value
Age Group		
<30	Ref	0.009
30-34	1.04 (0.93 to 1.29)	
35-39	1.13 (0.94 to 1.29)	
≥ 40	1.37 (1.13 to 1.65)	
Race group		
Non-Hispanic White	Ref	0.05
Non-Hispanic Black	1.17 (1.03 to 1.33)	
Non-Hispanic Asian	1.20 (0.97 to 1.49)	
Non-Hispanic other	1.15 (0.90 to 1.47)	
Hispanic	1.26 (1.05 to 1.52)	
Primary payer		
Government	Ref	0.24
Commercial	0.93 (0.82 to 1.04)	
Other	0.76 (0.48 to 1.20)	
Year		
2016	Ref	<0.001
2017	1.12 (0.96 to 1.31)	
2018	0.99 (0.85 to 1.16)	
2019	1.07 (0.91 to 1.25)	
2020	0.78 (0.66 to 0.92)	
Prior CD	0.79 (0.69 to 0.91)	<0.001
Chronic hypertension	1.03 (0.79 to 1.34)	0.83
Diabetes mellitus	0.77 (0.58 to 1.01)	0.06
Pre-eclampsia	1.33 (1.15 to 1.54)	<0.001
Admission APR severity of illness		
Mild	Ref	<0.001
Moderate	1.81 (1.61 to 2.04)	
Severe	4.55 (3.98 to 5.20)	
Extreme	21.60 (17.09 to 27.31)	
Pre-term delivery	0.92 (0.74 to 1.13)	0.42

Hospital was also included in the model as an independent variable. The *P* value for hospital was <0.001. Individual odds ratios for 33 hospitals within the state were not included in the table

AUROC for the model was 0.72,

*APR* all patient refined, *CD* Caesarean delivery

## Discussion

In a five-year, statewide observational cohort study that included over 100,000 CD patients, PPCs occurred in 1.7% of patients. PPCs were associated with both increased length of hospital stay and increased hospital charges. After adjusting for admission severity of illness and other potential confounders, Hispanic and Non-Hispanic Black patients were disproportionately impacted by PPCs. There also appeared to be considerable variation in the incidence of PPCs between hospitals, suggesting that the quality of obstetric care may vary considerably between hospitals.

In 2011, Maryland began to collect data on PPCs as part of its hospital acquired conditions program, which is distinct from the Centers for Medicare and Medicaid Services (CMS) hospital acquired conditions program. It is estimated that common hospital acquired conditions (e.g. pressure ulcers, surgical site infections, catheter associated urinary tract infections) increase healthcare spending in the Medicare program by approximately 150 million dollars per year, adding significant cost to United States healthcare [7]. Both CMS and Maryland penalize low-performing hospitals with a financial deduction of approximately 1% when hospital acquired conditions (i.e. PPCs) occur at a high rate in an individual hospital.

CD is the most common surgical procedure performed in the United States with over 500,000 patients per year having CD. There are few studies describing the incidence of PPCs after CD. Cardiovascular events are reported to occur in 0.2% of CD patients [8], infectious complications are reported to occur in 5-9% of patients [9–11], and VTEs are reported to occur in 0.3% of patients [12]. To our knowledge and based on our literature review, few studies have explored racial disparities in PPCs after CD. Prior studies have demonstrated that that Non-Hispanic Black patients and Hispanic patients are more likely to undergo primary CD, which puts them at greater risk for complications during childbirth [13–15]. Prior studies have also shown racial disparities in preterm birth rates [16, 17].

There are multiple potential causes of racial disparities in maternal and neonatal outcomes including poor access to antenatal care, disproportionate representation in low-quality hospitals, lack of adequate healthcare insurance, and a higher prevalence of comorbid conditions including diabetes mellitus, hypertension, and anemia [18, 19]. In the United States, differential access to high-quality hospitals is thought to be a major factor affecting healthcare outcomes with Non-Hispanic Black patients and Hispanic patients having less access to high-quality hospitals [20, 21]. Our data confirm that in Maryland the racial composition of patients differed dramatically by hospital, and that in hospitals with a high percentage of Hispanic patients there were more PPCs.

Our study highlights a continued need to address disparities in the quality of obstetric care provided in Maryland. Although PPCs may not be avoidable in every case, there are interventions that can reduce select PPCs, including surgical site infection. For example, appropriate antibiotic prophylaxis, appropriate preoperative skin preparation, use of clippers rather than razors, placental removal by traction rather than with manual removal, and suture closure of the subcutaneous tissue in deep wounds may all reduce surgical site infections after CD [3]. Device-related infections, such as catheter associated urinary tract infection, may be avoidable with early device removal and other best practices [22]. Standardizing perioperative procedures is an important aspect of ensuring quality and safety in surgical care. The implementation of Enhanced Recovery After Surgery (ERAS) protocols effectively eliminated racial disparities in postoperative length of stay among patients undergoing colorectal surgery [23]. Similarly, Enhanced Recovery After Cesarean (ERAC) delivery protocols may reduce or even eliminate racial disparities in PPCs after CD.

Future studies should explore why PPCs are more common in patients of different race-ethnicity. Contributing factors may include reduced access to high-quality hospitals, unconscious and conscious bias among healthcare workers, and differential access to regular antenatal care. Obstetric quality bundles and healthcare worker bias training may reduce racial disparities in PPCs and should be evaluated as potential interventions.

Our study’s principal strength is that it uses statewide validated data from Maryland’s HSCRC, which is used to assess hospital quality and adjust reimbursement. Our study also has limitations. First, clinical diagnoses were based on ICD codes rather than being entered by trained clinical personnel. Second, although we used APR severity of illness at admission to adjust for risk, this may not have been a complete risk adjustor. Third, our data are from a single state and hence they may not reflect practice throughout the United States. Fourth, race-ethnicity data were reported to the HSCRC by individual hospitals in Maryland and their practices for collecting this information may have been variable. Fifth, there may have been unobserved confounders that were not controlled for in our analysis. Finally, some PPCs may have been underreported in the HSCRC dataset.

## Conclusions

In summary, in a large observational cohort study, we found that PPC incidence is variable by hospital in Maryland and that Hispanic patients and Non-Hispanic Black patients were disproportionately impacted. Furthermore, we found that there was considerable variation in PPC incidence by hospital, suggesting that the quality of obstetric care differs by hospital. These findings highlight the continued need to address healthcare disparities through innovative programs in the United States. Also, further studies are needed to determine whether financial penalties for hospitals with a high PPC incidence leads to improvement in the quality of care or further harm for vulnerable groups who are disproportionately represented in these hospitals.

## Supplementary Information

**Additional file 1.**

## Data Availability

The datasets generated and/or analyzed during the current study are not publicly available because of the data use agreement that was signed to use the HSCRC data. However, the data can be obtained through a formal request and data use agreement with the HSCRC https://hscrc.maryland.gov/Pages/hsp-data-request.aspx. Information on how to obtain the data from the HSCRC can be obtained from Dr. Michael Mazzeffi (mimazzeffi@mfa.gwu.edu).

## Abbreviations

APR: all patient refined
CD: Cesarean delivery
CMS: centers for medicare and medicaid
ERAC: enhanced recovery after Cesarean
ERAS: enhanced recovery after surgery
HSCRC: health services cost review commission
ICD: international classification of disease
OR: odds ratio
PPC: potentially preventable complications
