# Supplementary material for: Racial-ethnic disparities in potentially preventable complications after cesarean delivery in Maryland: an observational cohort study
Source: BMC Pregnancy Childbirth. 2022 Jun 16;22:494. doi: 10.1186/s12884-022-04818-5 (PMC9204962; doi:10.1186/s12884-022-04818-5)
Supplement: Supplementary file 1 — Additional file 1. [file 12884_2022_4818_MOESM1_ESM.docx]

Supplemental Table 1. List of potentially preventable complications in grouper versions 36.0 and 37.0

| **PPC** | **Grouper category** |
| --- | --- |
| Shock | Extreme complication |
| Extreme central nervous system complications | Extreme complication |
| Acute pulmonary edema and respiratory failure with ventilation | Extreme complication |
| Ventricular fibrillation/cardiac arrest | Extreme complication |
| Renal failure with dialysis | Extreme complication |
| Post-procedural respiratory failure with tracheostomy | Extreme complication |
| Stroke and intracranial hemorrhage | Cardiovascular-respiratory complications |
| Acute pulmonary edema and respiratory failure without ventilation | Cardiovascular-respiratory complications |
| Pneumonia and other lung infections | Cardiovascular-respiratory complications |
| Aspiration pneumonia | Cardiovascular-respiratory complications |
| Pulmonary embolism | Cardiovascular-respiratory complications |
| Other pulmonary complications | Cardiovascular-respiratory complications |
| Congestive heart failure | Cardiovascular-respiratory complications |
| Acute myocardial infarction | Cardiovascular-respiratory complications |
| Other acute cardiac complications | Cardiovascular-respiratory complications |
| Peripheral vascular complications except venous thromboembolism | Cardiovascular-respiratory complications |
| Venous thrombosis | Cardiovascular-respiratory complications |
| Major gastrointestinal complications without transfusion or significant bleeding | Gastrointestinal complications |
| Major gastrointestinal complications with transfusion or significant bleeding | Gastrointestinal complications |
| Major liver complications | Gastrointestinal complications |
| Other gastrointestinal complications without transfusion or significant bleeding | Gastrointestinal complications |
| Post-procedural infection and deep wound disruption without procedure | Perioperative complications |
| Post-procedural wound infection and deep wound disruption with procedure | Perioperative complications |
| Reopening surgical site | Perioperative complications |
| Peri-operative hemorrhage and hematoma without hemorrhage control procedure or I and D procedure | Perioperative complications |
| Peri-operative hemorrhage and hematoma with hemorrhage control procedure or I and D procedure | Perioperative complications |
| Accidental puncture/laceration during invasive procedure | Perioperative complications |
| Post-procedural foreign bodies and substance reaction | Perioperative complications |
| Clostridium difficile colitis | Infectious complications |
| Cellulitis | Infectious complications |
| Moderate infections | Infectious complications |
| Septicemia and severe infections | Infectious complications |
| Urinary tract infection | Infectious complications |
| Catheter-related urinary tract infection | Infectious complications |
| Poisonings except from anesthesia | Malfunctions, reactions |
| Transfusion incompatibility reaction | Malfunctions, reactions |
| Iatrogenic pneumothorax | Malfunctions, reactions |
| Poisonings due to anesthesia | Malfunctions, reactions |
| Mechanical complication of device, implant, and graft | Malfunctions, reactions |
| Gastrointestinal ostomy complications | Malfunctions, reactions |
| Infection, inflammation, and other complications of devices, implants, or grafts except vascular infection | Malfunctions, reactions |
| Infection, inflammation, and clotting complications of peripheral vascular catheters and infusions | Malfunctions, reactions |
| Central venous catheter-related blood stream infection | Malfunctions, reactions |
| Medical and anesthesia obstetric complications | Obstetrical complications |
| Major puerperal infection and other major obstetric complications | Obstetrical complications |
| Other complications of obstetrical surgical and perineal wounds | Obstetrical complications |
| Genitourinary complications except urinary tract infection | Other medical and surgical complications |
| Renal failure without dialysis | Other medical and surgical complications |
| Diabetic ketoacidosis and coma | Other medical and surgical complications |
| Post-hemorrhagic and other acute anemia with transfusion | Other medical and surgical complications |
| In-hospital trauma and fractures | Other medical and surgical complications |
| Pressure ulcer | Other medical and surgical complications |
| Acute mental health changes | Other medical and surgical complications |
| Other surgical complication-moderate | Other medical and surgical complications |
| Encephalopathy | Other medical and surgical complications |
| Other complications of medical care | Other medical and surgical complications |
| Other in-hospital adverse events | Other medical and surgical complications |

PPC=potentially preventable complication
